# The Engineering Side of Hospital Work

**Published:** 1905-02-18

**Authors:** 


### THE ENGINEERING SIDE OF HOSPITAL WORK.

*Continued from page 358.*

### IV.—THE USE OF COLD STORAGE IN HOSPITALS.

The first question that arises, when the matter of cold storage comes up is, of what use is it for hospitals? The answer is, for many purposes, all of them leading either to increased economy, increased convenience, or increased income.

### In the Mortuary.

Foremost among possible uses of cold storage comes this. As hospital officials know, better than anyone else, there is a sad side to hospital work. While the healing work for which hospitals are instituted is constantly going on, there is a constant passage of some of its inmates "across the border." In many cases those who die have come from a distance. In all our large towns, but especially in London, there is a very large population that have come to the towns in the continual exodus from the country that is so marked of recent years, and often these have come from great distances. In many cases the first intelligence of the loss of some member of a family who has been from home for some time is a notice that he has died in a certain hospital. In all our great cities also, but especially in that huge aggregation of towns, Greater London, accidents are only too frequent, and too often result in death; and, again, sometimes the first intelligence of the accident is the notice of death. In all of these cases, and in many more that will occur to the readers of these articles, it is an inestimable boon for the relatives of those who have gone to their long home to be able to see them as they were immediately after death. If bodies are maintained at a low temperature, a little above freezing, the processes of decay are arrested, and the dead remain unchanged for as long as this state is maintained. This is, of course, pure sentiment, but hospitals exist only by reason of sentiment. It is the feeling for others that exists in the breasts of those who give, coupled with the feeling that they themselves may perhaps want the hospitality of one of those institutions that enables hospitals to exist, and everything that can encourage the feeling adds to the probable amount of the donations of the pious and charitable.

### As an Aid to Post-Mortems.

This is another important branch of hospital work in which cold storage can render great service. The process of decay sets in rapidly in many cases, unless it is arrested, and again it is low temperature that will arrest it. The possibilities of this branch of the service that cold storage can render are very great, and such as only a surgeon in busy practice can appreciate.

### The Preservation of Food.

Here we come to what should prove a considerable source of economy. All the hospitals the writer visited, that were not provided with cold stores, stated that their arrangements for feeding their patients were perfect. They knew exactly how much of everything was required, and their arrangements with the firms who catered for them were such that there could be no possible waste, as there could be nothing over. The tradesmen supplied exactly what was wanted, only in sufficient time before it was required for use, to enable it to be cooked, and all that was supplied was eaten. In this matter the experience of those hospitals appears to be very different from that of the large hotels, to which their arrangements must naturally tend. The hospital is after all the sick man's, principally the sick poor man's hotel, and it would appear on the face of it, as if what is true of hotels in the matter of the preservation of provisions must also be true of hospitals. Hotels find that by the aid of a proper system of cold stores, they are enabled to handle their provisions much better. They can buy better, when they can be sure of preserving any surplus, and there is no necessity to get rid of everything that has not been consumed. There is one substance which all hospitals use in large quantities for which a cold store is a great boon, viz., milk. The same rule appears to hold with regard to milk. Only the quantity that is known to be required is taken, and that is immediately consumed. But surely if this is possible with such things as meat, it is not possible with milk, and in hot weather milk turns very quickly, unless it is kept in an atmosphere artificially cooled. There are many other things that are consumed in hospitals in large quantities that would keep better in a cool atmosphere, and the question of economy must come in, and to a very large extent, where it is possible to preserve food that is consumed immediately. It should be understood that by the aid of cold stores almost every kind of perishable food may be preserved in good wholesome condition for periods that give the caterer a command of the markets and of his catering that he could not have without. The present writer has seen, in the cold store of an ocean steamer, a meat pie that had been to the West Indies and back, and was still as good as when it was first cooked.

Other Uses.

Among other uses for cold storage, and low temperatures generally, may be mentioned the fact that having steam, as all hospitals have, and refrigerating apparatus the surgeon and the dispenser have at their command a very wide range of temperature, the advantage of which can hardly be overestimated. For the dispenser the ability to command low temperatures at will without trouble would be a great boon, while it would also be of great service in enabling him to keep certain of his substances better in warm weather. In one dispensary the writer visited, he was shown a large tank of cod liver oil from which he understood there had been considerable waste with changes of temperature. As the dispenser explained to him, cod liver oil is composed of two distinct substances, one of which is a solid but is dissolved in the other. Above 70° F. or below 32° F. the solid is deposited from its solution and the cod-liver oil loses its virtue, or a considerable portion of it. With refrigeration apparatus on the ground, in addition to the steam already provided, it would be a simple matter to produce any temperature required within the oil tank. And the above is only one instance. Every chemist will be familiar with many cases where the command of a wide range of temperatures, especially low temperatures, would enable considerable economies to be effected. It should be understood that practically any temperature is obtainable from some degrees below zero F. upwards. It is only a matter of calculation and arrangement.

Apparatus Actually in Use in Hospitals.

Of the twelve hospitals in London visited by the writer five have cold storage plant. The most complete is at Middlesex, where one of Ransomes and Rapier's ammonia absorption apparatus is in use. It is capable of making 3 tons of ice in 24 hours. The evaporator coils are immersed in a brine tank a little way from the refrigerating plant, and brine is used to cool four chambers, one a mortuary for 12 bodies, two chambers for preserving food, and one for the specimens for the Pathological School. There is also an ice tank, making 30 cans, each of about 30 lbs. The engineer informed the writer that the cost of ice had been reduced since they had taken to making it themselves, from 22s. 6d. a ton to 8s. 3d. In addition to this they knew exactly what they were getting. The ice being made from condensed water taken from a part of the apparatus where the oil had been carefully extracted.

At St. Thomas's, one of Messrs. Haslam Foundry and Engineering Company's combined $CO_2$ compression apparatus is in use. It is driven by a 20 horse-power electric motor, supplied with current by the electric light service, and it cools a mortuary for 16 bodies, a cold store measuring 30′ × 20′ × 15′, and makes ice in an ice tank of 15 cells. In addition to this, it supplies cold brine to the milk steriliser. In this apparatus, which consists of a vessel similar to a churn, provided with a jacket, through which steam or cold brine can be forced. The milk is placed in the centre receptacle, steam being passed through the jacket, and the milk raised to a temperature of 150° F. It is kept at that temperature for 20 minutes, the milk being in motion the whole time, the steriliser being gently rotated by a belt from a shaft driven by an electro-motor. The steam is then exhausted from the jacket, and cold water run through it, the milk being cooled in this way to 85° or 90° F. This takes about another 20 to 25 minutes. Cold brine is then run through the jacket, from the brine tank of the refrigerator, and the temperature of the milk is then reduced to 40° F. This takes another 20 minutes. It is stated that after undergoing this process the milk will keep in the kitchen of the hospital for 48 hours quite sweet. The *rationale* is stated to be, the milk is heated sufficiently to kill pathogenic germs, and is then cooled quickly. The ability to keep—the time it will keep—is stated to depend upon the rapidity of cooling. The ice at St. Thomas's is also made from condensed water, and cooling by means of brine circulation is used.

At St. George's, London, and University, Messrs. J. and E. Hall's $CO_2$ apparatus is employed. At St. George's there is a small combined plant, barely sufficient for the mortuary of 12 bodies, for which it is used. The mortuary is divided into four cells, the whole being cooled by a brine grid passing across the top. The compression plant, which is fixed in a chamber close to the mortuary, and to the mortuary chapel, is driven by an electric motor, supplied with current from the electric lighting service.

At the London Hospital there is a 1-ton plant, driven by a 13-h.p. electric motor, the evaporating tank being separate, and the cooling water for the condenser being cooled by a spray cooling tank on the roof. The apparatus cools a mortuary for 16 bodies, and for an ice tank of 40 cans, of $\frac{1}{2}$ cwt. each. There is also an ice store under the compressor chamber, capable of storing 20 tons of ice. The arrangement of the cooling apparatus mentioned above is as follows:—There is a shallow tank, exposing a large surface to any wind or sun there may be. In addition, there are a number of nozzles distributed over the surface of the water, supplied by pipes connected with them, and through the nozzles the water to be cooled is continually sprayed, the result being that a certain amount of evaporation from the surface of the water and from the spray takes place, the evaporation cooling the whole of the water.

At University Hospital, there is a combined plant driven by a horizontal steam-engine, supplying a mortuary for eight bodies, and two cold chambers for food, as well as an ice tank having eight cans of 17 lbs. of ice each.

There are two points the writer would mention before concluding this article. He fears that the question of pure ice is hardly appreciated in hospitals. Making ice from condensed water, taken from the exhaust-steam, is good, but it is hardly sufficient. The modern practice is to distil, skim, and reboil and filter the water before using it for making ice, the object being to remove every foreign substance, and all possible germs.

The other point is, the use of the cold store, even where it is regularly employed, is not always fully understood, nor the fact that the air of the cold chamber can be, and is, in modern cold stores, continually renewed, and the air that enters the cold chamber purified, and dried where necessary, before it is allowed to enter. It should be understood that the refrigeration engineer, if his apparatus is properly constructed and properly run, has complete control of all that is in the cold chamber, and of the air supplied to it.

